# A Novel *CDH1* Variant Identified in a Chinese Family with Blepharocheilodontic Syndrome

**DOI:** 10.3390/diagnostics12122936

**Published:** 2022-11-24

**Authors:** Bichen Lin, Yang Liu, Lanxin Su, Hangbo Liu, Hailan Feng, Miao Yu, Haochen Liu

**Affiliations:** 1 First Clinical Division, Peking University School and Hospital of Stomatology & National Center of Stomatology & National Clinical Research Center for Oral Diseases & National Engineering Research Center of Oral Biomaterials and Digital Medical Devices, Beijing 100081, China; 2 Department of Prosthodontics, Peking University School and Hospital of Stomatology & National Center of Stomatology & National Clinical Research Center for Oral Diseases & National Engineering Research Center of Oral Biomaterials and Digital Medical Devices, Beijing 100081, China

**Keywords:** *CDH1*, Blepharocheilodontic syndrome, gene variant, tooth agenesis

## Abstract

The goal of the current study was to identify the pathogenic gene variant in a Chinese family with Blepharocheilodontic (BCD) syndrome. Whole-exome sequencing (WES) and Sanger sequencing were used to identify the pathogenic gene variant. The harmfulness of the variant was predicted by bioinformatics. We identified a novel heterozygous missense variant c.1198G>A (p.Asp400Asn) in the *CDH1* gene in the proband and his mother with BCD syndrome. The sequencing results of three healthy individuals in this family are wild type. This result is consistent with familial co-segregation. According to ReVe, REVEL, CADD, gnomAD, dbSNP, and the classification of pathogenic variants with the standards of the 2015 American College of Medical Genetics and Genomics and the Association for Molecular Pathology (ACMG), c.1198G>A (p.Asp400Asn) is predicted to be a likely pathogenic. We observed that variant c.1198G>A (p.Asp400Asn) was located in the extracellular cadherin-type repeats in CDH1. Amino acid sequence alignment of the CDH1 protein among multiple species showed that Asp400 was highly evolutionarily conserved. The conformational analysis showed that this variant might cause structural damage to the CDH1 protein. Phenotypic analysis revealed unique dental phenotypes in patients with BCD syndrome, such as oligodontia, conical-shaped teeth, and notching of the incisal edges. Our results broaden the variation spectrum of BCD syndrome and phenotype spectrum of CDH1, which can help with the clinical diagnosis, treatment, and genetic counseling in relation to BCD syndrome.

## 1. Introduction

Tooth agenesis is one of the most common congenital malformations in humans, which causes clinically challenging problems, such as malocclusion, masticatory dysfunction, speech changes, and aesthetic problems [1]. The prevalence of tooth agenesis is approximately 2–10% in different regions of the population [2,3,4]. Tooth agenesis may appear as part of a recognized genetic syndrome or as a non-syndromic familial form, which occurs as an isolated trait. Tooth agenesis has been associated with many factors, including genetics, epigenetics, and environmental factors. Among them, genetic defects have been demonstrated to play a major role [1].

Blepharocheilodontic (BCD) syndrome (OMIM 119580) is a rare autosomal dominant disorder characterized by eyelid malformations, cleft lip/palate (CL/P), and dental anomalies [5,6]. Eyelid anomalies (i.e., ectropion, euryblepharon, lagophthalmy, and distichiasis) are typical symptoms [7]. Bilateral CL/P is also one of the main features [8]. Dental anomalies usually manifest as oligodontia and a conical tooth shape [5]. In addition, hypothyroidism, hair abnormalities, dysmorphic facial features, syndactyly, imperforate anus, dermoid cysts, and neural tube defects were also reported in a few cases [5,9,10].

Recently, studies have identified that the pathogenic genes of BCD syndrome are *cadherin 1* (*CDH1*) and *catenin delta 1* (*CTNND1*) [7,8]. *CDH1* encodes E-cadherin, a single-pass transmembrane protein playing an essential role in epithelial cell adherence [11]. *CTNND1* encodes catenin delta 1 (alias p120ctn), an armadillo repeat-containing protein that interacts with the juxta-membrane cytoplasmic tail of CDH1 and controls the stability of the complex [12]. Studies on mouse models presented the significant roles of *CDH1* in eyelid, craniofacial, tooth, and hair developments [13,14,15,16]. Additionally, *CDH1* gene variants were also reported to be associated with diffuse gastric and lobular breast cancer syndrome with CL/P (HDGC; OMIM 137215) or non-syndromic CL/P (NSCLP) [17,18,19,20,21]. In 2016, Nishi et al. identified a de novo *CDH1* missense variant in a patient with symptoms similar to BCD syndrome [22]. In 2017, Ghoumid et al. identified five *CDH1* deleterious missense variants and three *CTNND1* truncating variants in 11 patients with BCD syndrome from 8 families and first pointed out that *CDH1* and *CTNND1* are the pathogenic genes of BCD syndrome [7]. In 2018, Kievit et al. further confirmed that *CDH1* and *CTNND1* are pathogenic for BCD syndrome, and they identified *CDH1* or *CTNND1* variants in 15 patients with BCD syndrome [8].

In this study, we performed whole-exome sequencing (WES) and clinical assessments of a family with inherited BCD syndrome to investigate the genetic causes and clinical phenotypes. A novel missense *CDH1* variant was identified in the family. Then, we use bioinformatic and conservation analyses to predict the pathogenicity of the identified variant. In addition, we analyzed the number and morphological variation of teeth in patients with BCD syndrome.

## 2. Materials and Methods

### 2.1. Subjects

The proband and his family were recruited at the Department of Prosthodontics, Peking University School of Stomatology. Oral and maxillofacial examinations were performed by experienced dentists. Panoramic radiographs were taken to confirm the position of missing teeth. Eye and hair examinations were performed by ophthalmologists and dermatologists, respectively. Blood samples were provided by two patients and three unaffected family members. Informed consent was obtained from all the participants, and the proband provided consent for the publication of the intraoral clinical photographs. This study was conducted with permission from the Ethics Committee of Peking University School and Hospital of Stomatology (PKUSSIRB-202162021).

### 2.2. WES

Peripheral blood genomic DNA was extracted using the BioTek DNA Whole-Blood Mini Kit (BioTek, Beijing, China), referring to the instructions. WES was performed in all the five available family members by Beijing Angen Gene Medicine Technology (Beijing, China) using the Illumina-X10 platform to identify the potential pathogenic gene variants. Based on the WES results, orodental-related genes were annotated [23]. Then, we filtered all the nonsynonymous single-nucleotide variants and insertions/deletions according to the MAF ≤ 0.01 in the databases, including the single-nucleotide polymorphism database (dbSNP, http://www.ncbi.nlm.nih.gov/projects/SNP/snp_summary.cgi , accessed on 1 October 2020) and the genome aggregation database (gnomAD, http://gnomad.broadinstitute.org accessed on 1 October 2020). The variants that affected protein function were predicted based on the results obtained from ReVe [24], rare-exome-variant ensemble learner (REVEL) [25], and combined annotation-dependent depletion (CADD) [26].

### 2.3. Sanger Sequencing

To verify the variants and analyze the familial co-segregation, polymerase chain reaction (PCR) was employed to amplify the exon 9 of the *CDH1* gene (NM_004360) (primers can be provided according to the requirements). The PCR products were sent to Beijing Angen Gene Medicine Technology for purification and Sanger sequencing.

### 2.4. Conservation Analysis

For conservation analysis, the amino acid sequences of CDH1 among different species were obtained from the NCBI database (https://www.ncbi.nlm.nih.gov/, accessed on 30 September 2022). Molecular Evolutionary Genetics Analysis Version 11 (MEGA 11.0) [20] was used to conduct the multiple sequence alignment.

### 2.5. Conformational Analysis

The three-dimensional structure of the CDH1 protein was predicted using the Alpha Fold Protein Structure Database (https://alphafold.ebi.ac.uk/, accessed on 1 October 2020). PyMol v2.1 (Molecular Graphics System, DeLano Scientific, San Carlos, CA, USA) was used to analyze the three-dimensional structural changes caused by the variant.

## 3. Results

### 3.1. Clinical Findings

**Figure 1:**
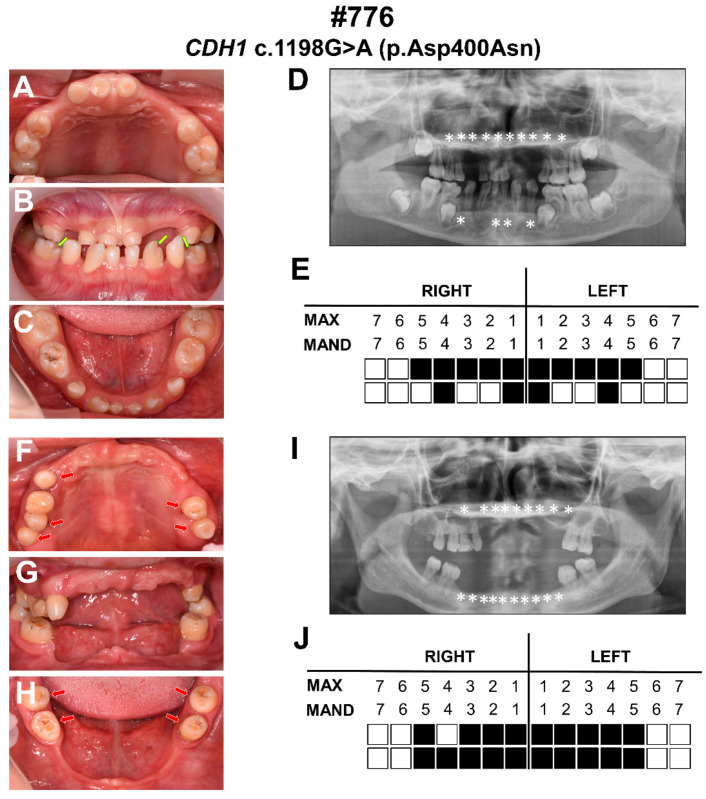
Clinical photographs and panoramic radiographs of the proband and his mother. (**A**–**C**) Intro-oral photographs of the proband. (**D**) Panoramic radiograph of the proband. (**E**) Schematic of congenital missing permanent teeth of the proband. (**F**–**H**) Intro-oral photographs of the proband’s mother. (**I**) Panoramic radiograph of the proband’s mother. (**J**) Schematic of congenital missing permanent teeth of the proband’s mother. Asterisks and solid squares indicate congenitally missing teeth; the yellow arrows indicate teeth with notching of the incisal edges; the red arrows indicate conical-shaped teeth. Max: maxillary; Mand: mandibular.

This was a three generation family, in which the proband and his mother were BCD patients. The proband was a 9-year-old boy who presented with congenital missing of all primary teeth, congenital missing of 14 permanent teeth, abnormalities in tooth morphology, euryblepharon, ectropion, distichiasis, sparse hair, high frontal hairline, and broad forehead (Figure 1A–E and Table 1). The abnormality in the morphology of the teeth was the notching of the incisal edges. His mother was a 35-year-old female who presented with congenital missing of all primary teeth, congenital missing of 19 permanent teeth, abnormalities in tooth morphology, euryblepharon, ectropion, distichiasis, sparse hair, high frontal hairline, and broad forehead (Figure 1F,G and Table 1). The abnormality in the morphology of the teeth was that they were conical-shaped.

### 3.2. The Identification of the CDH1 Variant

**Figure 2:**
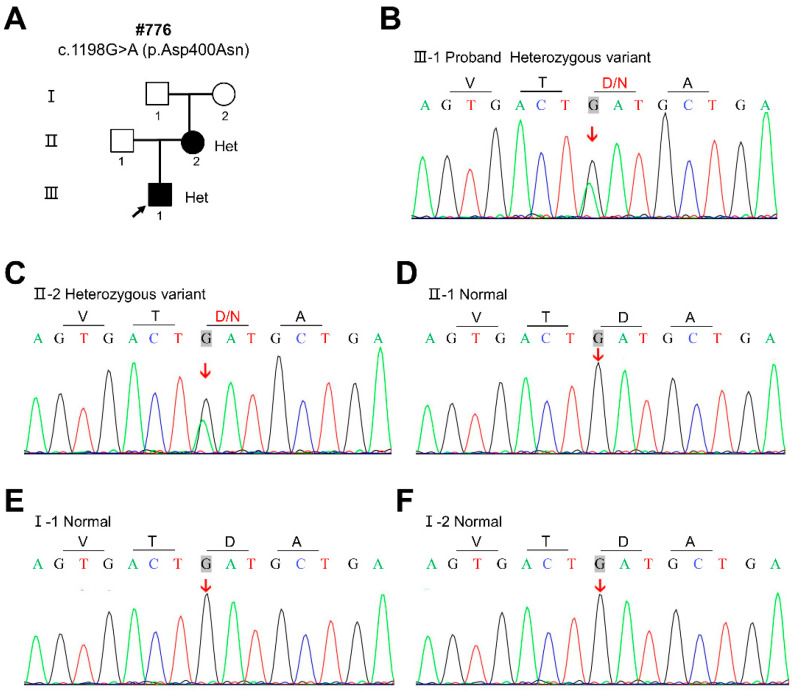
Family pedigree and sequencing chromatograms of the proband and family members in this study. (**A**) Pedigree of the BCD family #776. (**B**) Sequencing chromatograms showing a heterozygous *CDH1* variant c.1198G>A (p.Asp400Asn) in the proband (III-1). (**C**) Sequencing chromatograms showing a heterozygous *CDH1* variant c.1198G>A (p.Asp400Asn) in the proband’s mother (II-2). (**D**–**F**) Sequencing chromatograms showing a wild-type *CDH1* genotype in the three healthy individuals (I-1, I-2, and II-1).

By WES and Sanger sequencing, we identified a novel heterozygous missense variant c.1198G>A (p.Asp400Asn) in the *CDH1* gene (NM_004360) in the proband and his mother (Figure 2B,C). According to ReVe, REVEL, CADD, gnomAD, dbSNP, and the classification of pathogenic variants with the standards of the 2015 American College of Medical Genetics and Genomics and the Association for Molecular Pathology (ACMG), c.1198G>A (p.Asp400Asn) was predicted to be likely pathogenic (Table 2). The sequencing results of three healthy individuals (Ⅰ-1, Ⅰ-2, and Ⅱ-1) in this family are wild type (Figure 2D–F). This result is consistent with familial co-segregation.

### 3.3. Conservation and Conformational Analyses

**Figure 3:**
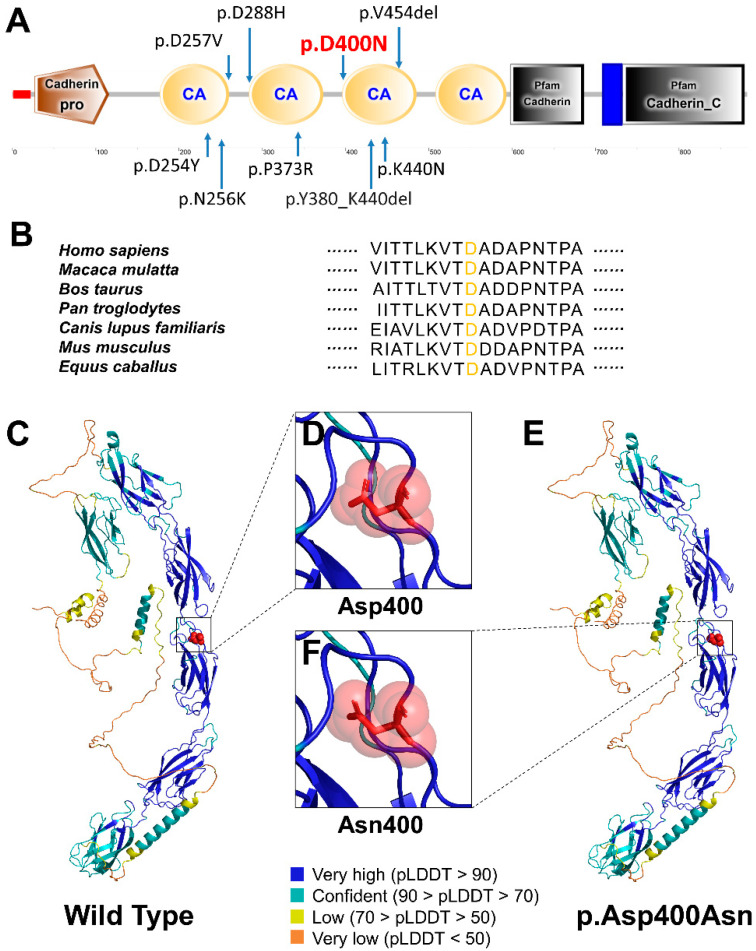
Location and conservation and conformational analyses of CDH1 variant p.Asp400Asn. (**A**) Schematic diagram of the wild-type human CDH1 (NP_004351.1) protein and the distribution of the novel CDH1 variant identified in this study and reported variants identified in patients with BCD syndrome. (**B**) Conservation analysis of the affected amino acids in CDH1 protein. (**C**) Tertiary structure of the wild-type CDH1 protein predicted by AlphaFold. (**E**) Tertiary structure of the p.Asp400Asn variant CDH1 protein. (**D**,**F**) Predicted conformational consequences of CDH1: p.Asp400Asn.

We conducted the conservation and conformational analyses to predict the effects of the identified novel *CDH1* variant c.1198G>A (p.Asp400Asn). This variant resulted in the replacement of Asn at position 400 of CDH1 protein (NP_004351.1) with Asp. We observed that c.1198G>A (p.Asp400Asn) was located in the extracellular cadherin-type repeats in CDH1 (Figure 3A). The amino acid sequence alignment of the CDH1 protein among multiple species showed Asp400 was highly evolutionarily conserved (Figure 3B). The residue at sequence position 400 in this protein is an aspartic acid that has a negatively charged side chain, making it hydrophilic (i.e., preferring the surface of the protein to its interior) (Figure 3C,D). The variant residue is an asparagine that has a neutral side chain (Figure 3F,E).

## 4. Discussion

BCD syndrome is an extremely rare autosomal dominant genetic disease. Its main features are cleft lip/palate, eye-lid malformations, and ectodermal dysplasia. Few BCD cases have been reported in the Asian population; there was only one literature report on a Japanese girl who had a cleft lip and palate, choanal atresia, tetralogy of Fallot, and a neural tube defect, which was caused by a missense variant in the *CDH1* gene [22]. This girl’s symptoms were similar to BCD syndrome, but unfortunately the authors did not describe the dental phenotypes associated with the symptoms.

In the present study, the proband and his mother were congenitally missing 14 and 19 permanent teeth, respectively, and both had missing primary teeth. The abnormality in the morphology of their teeth included conical-shaped teeth and teeth with the notching of the incisal edges. These phenotypes did not appear simultaneously in patients with tooth agenesis in our previous study [27,28,29,30,31,32]. Awadh et al. described the dental characteristics of patients with BCD syndrome in detail: the mean number of missing permanent teeth was 13.7; the location of missing teeth included maxillary central incisors, maxillary lateral incisors, maxillary canines, and maxillary premolars; teeth morphological abnormalities included conical-shaped teeth, taurodontism, and notching of the incisal edges [33]. Thus, the dental characteristics of the patients with BCD syndrome in our study were consistent with the study conducted by Awadh et al. [33]. Moreover, eye symptoms in this study included euryblepharon, ectropion, and distichiasis, which were also consistent with the diagnosis of BCD syndrome [5,34]. The patients in this study also had other symptoms of BCD syndrome, such as sparse hair, a high frontal hairline, and broad forehead. Although most of the reported patients with BCD syndrome have a bilateral cleft lip and palate [5,35], the patients in this study did not. By WES and Sanger sequencing, we segregated a novel heterozygous missense variant c.1198G>A (p.Asp400Asn) in the *CDH1* gene in the proband and his mother. The sequencing results of three healthy individuals (Ⅰ-1, Ⅰ-2, and Ⅱ-1) in this family are wild type. This result is consistent with family co-separation. In 2017, Ghoumid et al. first reported that BCD syndrome was a *CDH1* pathway-related disorder due to variants in *CDH1* and *CTNND1*, and identified five *CDH1* deleterious missense variants and three *CTNND1* truncating variants in 11 patients with BCD syndrome from eight families [7]. In 2018, Kievit et al. identified *CDH1* or *CTNND1* variants in 15 patients with BCD syndrome, which further confirmed the relationship between *CDH1* and BCD syndrome [8]. Combining the clinical phenotype and genetic test results, we diagnosed the patients in this study with BCD syndrome caused by the *CDH1* variant.

The *CDH1* gene encodes a classical cadherin of the cadherin superfamily. Five cadherin-type repetitions are joined together by Ca^2+^ ions to create rigid, rod-like proteins in the extracellular region of the CDH1 protein. In prior studies, all BCD-associated missense variations were found at highly conserved extracellular cadherin-type repeats of CDH1, which directly interact with Ca^2+^ ion [7,8]. In this study, the variant CDH1:p.Asp400Asn was also found in extracellular cadherin-type repeats. This result is consistent with the previous studies.

It has been previously reported that *CDH1* gene variants may lead to hereditary diffuse gastric cancer (HDGC) [21], with or without CL/P, and invasive lobular breast cancer (ILC) [36]. The patients in this study did not have any history of cancer, and no CLP was detected. Ghoumid et al. concluded the phenotype spectrum of *CDH1* variants is extensive, including asymptomatic carriers, NSCLP, HDGC with or without CL/P, BCD syndrome, etc. [7]. The phenotype of *CDH1*-associated BCD syndrome also varied widely. The patients in this study had dental and ocular symptoms, but not symptoms such as CLP, lagophthalmos, ankyloblepharon, malformed ears, everted lower lip, hypothyroidism, hypertelorism, imperforate anus, dermoid cysts, and neural tube defect. It has previously been reported that the *CDH1* missense variant is likely to induce cell-specific biological behavior with distinct clinical impacts [37]. However, the specific pathogenic mechanisms require further study.

The tooth phenotype associated with BCD syndrome is noteworthy. The dental symptoms of the patients in this study included the congenital missing of primary and permanent teeth and abnormalities in the morphology of their teeth. The number of missing permanent teeth exceeded six (except for the third molar), which could be diagnosed as oligodontia. The patients’ molars and premolars had small, tapered crowns. In addition, the notching of the incisal edges was also a unique feature. At present, only the study conducted by Awadh et al. [33] described in detail the dental abnormalities of patients with BCD syndrome, which is consistent with the present study. These characteristic dental phenotypes could be used as a basis for the diagnosis of BCD syndrome in future research.

Dental development in humans is a lengthy and intricate process involving a series of reciprocal and sequential interactions between the embryonic oral epithelium and the neural crest-derived mesenchyme. Despite environmental interference, tooth development is mainly controlled by genes. So far, over 200 genes have been identified during tooth development, and these genes are involved in multiple signaling pathways, including the ectodysplasin A (EDA), bone morphogenetic protein (BMP), sonic hedgehog homolog (SHH), fibroblast growth factor (FGF), wingless-type MMTV integration site family (WNT), and transform growth factor (TGF) pathways [38]. Variants in any of these strictly balanced signaling cascades can lead to tooth agenesis and/or other organ defects. The causative gene for non-syndromic tooth agenesis has been a difficult area of research, and there are still many patients with undetected causative genetic variants. Extensive research of publicly available datasets identified 15 genes responsible for non-syndromic tooth agenesis, and genotype–phenotype association analysis indicated that the majority of these genes are also responsible for syndromic tooth agenesis or other diseases [39]. Therefore, the causative genes for non-syndromic tooth agenesis were found from syndromic tooth agenesis. The phenotypes of the different variants of *CDH1* were extremely different. As a newly identified syndromic tooth agenesis causative gene, *CDH1* can serve as a potential causative gene for non-syndromic tooth agenesis.

## 5. Conclusions

In summary, we reported a novel heterozygous missense variant c.1198G>A (p.Asp400Asn) in the *CDH1* gene in a Chinese family with BCD syndrome. This is the first report of *CDH1*-associated BCD syndrome in the Chinese population. Our study analyzed the pathogenicity of this variation and summarized the characteristic tooth morphological variation of BCD syndrome. In addition, we also suggested that CDH1 can be used as a potential non-syndromic tooth agenesis pathogenic gene for screening. Our results broaden the variation spectrum of BCD syndrome and phenotype spectrum of CDH1, which could help with the clinical diagnosis, treatment, and genetic counseling of the disease. However, the pathogenic mechanism of *CDH1*-associated BCD syndrome remains unclear and needs to be studied more extensively.

## Figures and Tables

**Table 1:** Clinical features of patients in this study.

Clinical Features	The Proband	Ⅱ-2
Gender	Male	Female
Age	9	35
Number of missing permanent teeth	14	19
Congenital missing of primary teeth	+	+
Abnormality in morphology of teeth	+	+
CL/P	-	-
Euryblepharon	+	+
Lagophthalmos	-	-
Ectropion	+	+
Lacrimal duct abnormalities	-	-
Distichiasis	+	+
Ankyloblepharon	-	-
Sparse hair	+	+
High frontal hairline	+	+
Broad forehead	+	+
Malformed ears	-	-
Everted lower lip	-	-
Hypothyroidism	-	-
Hypertelorism	-	-
Imperforate anus	-	-
Dermoid cysts	-	-
Neural tube defect	-	-

**Table 2:** Pathogenic prediction of the *CDH1* variant identified in this study.

Patients	Variant	Variant Type	Domain	ReVe	REVEL	CADD	dbSNP/ gnomAD	ACMG Classification (Evidence of Pathogenicity)
#776 III-1; #776 II-2	c.1198G>A/p.Asp400Asn	Hetero-zygous	3rd CA	0.680 Disease causing	0.643 Disease causing	26.3 Disease causing	-	Likely pathogenic PS2, PM2, PP3, PP4

PS2: De novo in a patient with the disease but no family history. PM2: Absent from controls in Exome Aggregation Consortium, Exome Sequencing Project, or 1000 Genomes Project. PP3: Multiple lines of computational evidence support the existence of a detrimental impact on the gene or gene product. PP4: The phenotypic or family history of a patient is very precise for a disease with a single genetic cause.

## Data Availability

The variations identified in this study were submitted to the ClinVar database (submission ID SCV000979048.2).
